# Comparison of Cytopathology Yield of Fine-Needle Aspiration Biopsy Using 25G Versus 27G Needles for Melanocytic Uveal Tumors †

**DOI:** 10.3390/jcm14113650

**Published:** 2025-05-23

**Authors:** Gustavo Rosa Gameiro, Carolina C. Valente, James J. Augsburger, Zelia M. Correa

**Affiliations:** 1 Department of Ophthalmology, Bascom Palmer Eye Institute, University of Miami Miller School of Medicine, Miami, FL 33136, USA; gustavo.gameiro@med.miami.edu; 2 Centro Universitario Lusiadas, Faculdade de Ciencias Medicas de Santos, Santos 11045-101, SP, Brazil; carolinacorreavalente@gmail.com; 3 Ocular Oncology Service, Department of Ophthalmology, College of Medicine, University of Cincinnati, Cincinnati, OH 45221, USA; augsbujj@uc.edu; 4 Department of Ophthalmology, Ocular Oncology Service, Bascom Palmer Eye Institute and Sylvester Comprehensive Cancer Center, University of Miami Miller School of Medicine, Miami, FL 33136, USA

**Keywords:** melanoma, uveal neoplasms, needle biopsy, cytology, ocular oncology, biopsy

## Abstract

**Background/Objectives**: This study aims to evaluate whether fine-needle aspiration biopsy (FNAB) of melanocytic uveal tumors (MUTs) using 27-gauge (27G) needles yields aspirates like those obtained using 25-gauge (25G) needles for cytology. **Methods**: A retrospective review was conducted on 32 primary uveal melanomas (PUMs). Tumors were sampled at three adjacent sites, first using a 27G needle for gene expression profile (GEP) testing, second and third with 27G and 25G needles for cytology. The endpoints evaluated were the sufficiency of aspirates for cytopathology and GEP. **Results**: Among the 32 patients, 17 tumors were choroidal, 6 ciliochoroidal, 7 iridociliochoroidal, and 2 exclusively iridic. Tumor diameter ranged from 3.3 mm to 23 mm (mean 13.2 mm), and thickness ranged from 0.5 mm to 12 mm (mean 6.4 mm). Aspirates from both needle sizes were sufficient for cytopathological diagnosis and GEP in 31 of 32 cases (96.9%). The single insufficient aspirate was insufficient with both the 27G and 25G needles. The cytopathology was identical in all other cases. The tumors were Class 1 in 22 cases (71.0%) and Class 2 in 9 cases (29.0%). **Conclusions**: FNAB aspirates of MUTs using 27G needles appear sufficient for cytology and GEP in most cases, showing a similar diagnostic yield compared to 25G needles.

## 1. Introduction

In most centers in the US, the current management of uveal melanocytic tumors clinically diagnosed as a uveal melanoma (UM) or an indeterminate melanocytic lesion (encompassing atypical nevi and large nevi versus small melanomas, i.e., uveal melanocytic tumor) includes a prognostic, confirmatory, or diagnostic biopsy prior to or at the time of treatment [1,2].

The diagnostic and prognostic assessment of these tumors allows appropriate and timely treatment and subsequent individualized oncologic care and surveillance testing [2]. Several techniques are currently being used to biopsy intraocular tumors, in particular, uveal melanocytic tumors. These techniques include transscleral, transaqueous (or translimbal) as well as multiple transvitreal approaches, using either a needle or a vitrector [3,4,5].

Because of the challenge of yielding aspirates that are sufficient for diagnosis while minimizing the procedure-associated risks that include bleeding and retinal detachment, there is a continuous search to improve biopsy techniques to be less invasive [1]. While using a smaller gauge needle may reduce the risks of complications, thicker needles will retrieve larger samples at the cost of tissue disruption [5,6]. This balance between successful specimen procurement and minimizing complications led us to consider transitioning from using 25-gauge (25G) (our standard of care at the time) [3] to 27-gauge (27G) needles. To address the concern of decreased biopsy yield, we decided to harvest sequential samples using 27G and 25G needles and compare the findings as a quality measure before changing our institutional standard of care so not be detrimental to patients.

Recent publications have shown that fine-needle aspiration biopsy (FNAB) carries limited risks that must be balanced with the need for obtaining adequate specimen yield [2,5,6]. Despite this evidence supporting the safety and benefit of tumor biopsy, there are still those who resist to adopt such important tool claiming reasons such as risk of vision threatening complications, low diagnostic yield of such procedures, and risk of tumor spreading [1,3,4,5,7].

The most common observed complications are small subretinal or vitreous hemorrhage close to the biopsy site, rhegmatogenous retinal detachment is less uncommon and a virtual chance of tumor-seeding. In this sense, McCannel et al. (2012) reported no increased risk for metastasis due to FNAB when using repeated sampling of the tumor not controlling for variables such as tumor location, depth of the needle, exposure of the orbit by washing the ocular surface with distilled water, and sterilization of the needle tract with cryotherapy [7].

Some previous studies have examined the use of 25G FNAB needles in intraocular tumor biopsies. Singh et al. (2016) reported a 92% diagnostic yield using 25G FNAB needles [8], while two studies by Kim et al. (2016 and 2018) corroborated that FNAB is a safe procedure, providing sufficient cytopathological yields for subsequent cytologic and genomic analysis without tumor dissemination [9,10]. However, these studies did not compare 25G and 27G needles.

To address this important technical dilemma, the purpose of this study is to review the yield, quality of the cytology sample, and complications associated with 25G and 27G needles utilized for FNAB of melanocytic uveal tumors in a single center.

## 2. Materials and Methods

This was a retrospective review of consecutive patients with a clinical diagnosis of UM evaluated by FNAB for diagnostic, confirmatory or prognostic purposes at the Department of Ophthalmology, University of Cincinnati College of Medicine, Cincinnati, OH. In the senior author’s practice, FNAB has been part of the standard of care as diagnostic, confirmatory, or prognostic since 2007. The data were abstracted in a de-identified manner and covered by a waiver provided by the Institutional Review Board to abstract data from the ocular oncology database. Prior to the procedure, the patients were informed of this change in practice and voluntarily agreed to having their tumor sampled by two different gauge needles, understanding the risks involved and subsequently providing a signed consent form for the procedure. The Declaration of Helsinki and the Health Insurance Portability and Accountability Act were followed throughout the study.

**Figure 1:**
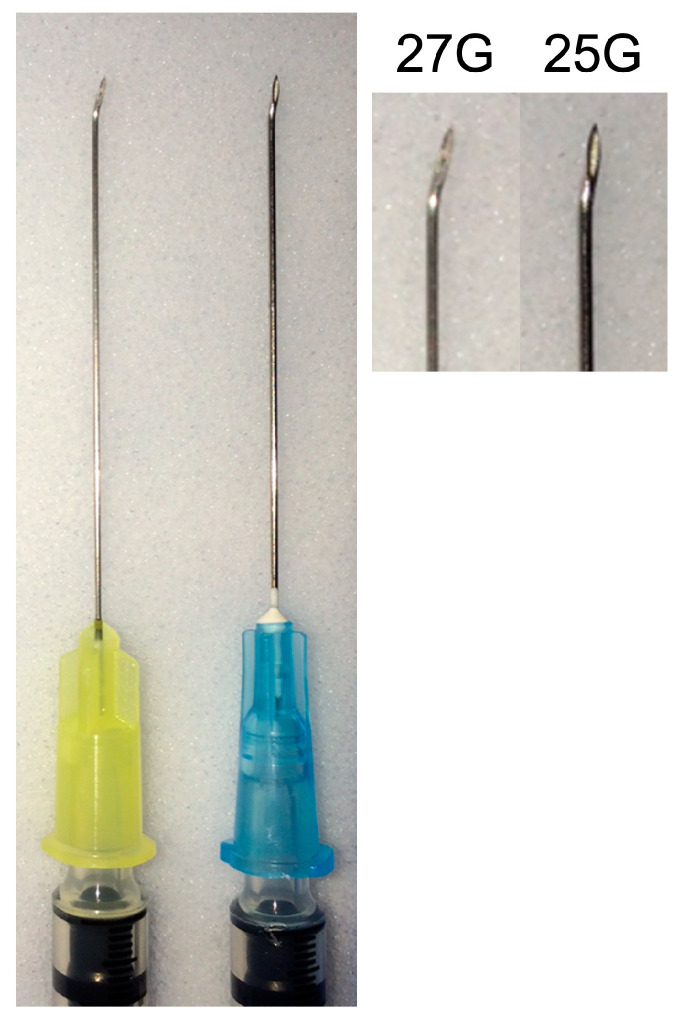
Side-by-side images of 27-gauge and 25-gauge needles utilized for fine-needle aspiration biopsy.

The biopsy technique employed was previously described [11]. Briefly, it was an outpatient procedure conducted in the operating room with local anesthesia (via retrobulbar injection). For small (less than 1.5 mm thick) posterior tumors, the procedure involved an eye-wall puncture in the pars plana region determined based on the tumor’s position within the eye. Preparation for the site involved creating a partial-thickness scleral incision, about 0.5 to 1.0 mm long, parallel to the limbus at a precise distance of 3.5 mm from it. The needle was bent slightly at the end of its bevel using a hemostat or a large needle driver, with the bending angle tailored to the tumor’s intraocular location (Figure 1). The eye was stabilized using two or more 4-0 black silk traction sutures placed behind the rectus muscles’ insertions. PUMs with a thickness over 1.5 mm were biopsied in a similar fashion without bending the needle tip.

During the biopsy procedure, the needle’s position was meticulously monitored with indirect ophthalmoscopy as it passed through the vitreous and retina into the choroidal tumor. Once the needle penetrated the tumor, aspiration was performed with subtle rotating movements and repeated aspiration cycles. The needle was promptly removed after the aspirations, and light pressure was applied to the puncture site to ensure hemostasis for one minute.

**Figure 2:**
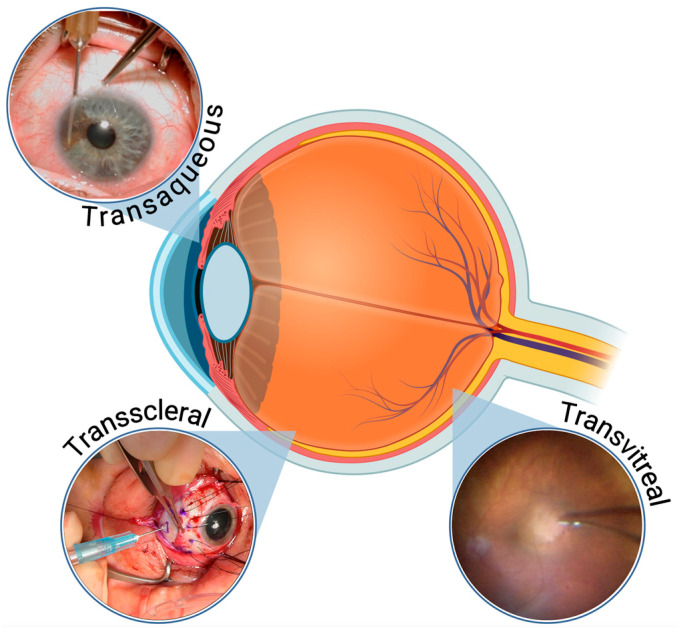
FNAB techniques for uveal melanomas. Transaqueous (**top left**): for anterior tumors (iris/ciliary body) via the anterior chamber. Transscleral (**bottom left**): for equatorial/anterior choroidal tumors via the sclera. Transvitreal (**bottom right**): for posterior tumors via the vitreous cavity.

For posterior tumors at or anterior to the equator, a scleral tunnel (2 mm long) was created over the identified tumor epicenter, through which the needle was advanced to obtain the specimen. For tumors in the iris, a limbal approach was taken progressing the needle into the tumor under direct visualization using a microscope. In these cases, shorter needles were used. The aspiration was carried out in a similar fashion regardless of the tumor location. At the end of the biopsy, the puncture site was thoroughly rinsed with distilled water, and double freeze-thaw cryotherapy was used to sterilize the aspiration pathway. Figure 2 below illustrates the FNAB techniques by tumor location.

In our protocol, each tumor underwent sampling of three distinctly located sites. For the initial site, a 27G needle was employed. The aspirate collected from this site was specifically designated for the DecisionDx-UM^®^ gene expression profile test (Castle Biosciences, Inc., Friendswood, TX, USA) for prognostic classification. The second site was also sampled using a 27G needle, mirroring the technique used for the first site. However, this sample was sent for cytological analysis. For the third and final site, a 25G needle was utilized. The aspirate from this site, like the second one, was dedicated to cytological examination and compared to the previous one.

These aspirates were flushed on a Poly-l-Lysine-coated glass slide (Thermo Fisher Scientific, Inc., Waltham, MA, USA—Product No. P4981), and smeared using a second slide. The specimen was fixated with alcohol spray (Azer Scientific Inc., Morgantown, PA, USA) and air dried for 15 min. After that, the slides were stained according to a standard hematoxylin and eosin (H&E) protocol.

This approach allowed us to compare the quality and diagnostic yield between the samples obtained with needles of different calibers (25G vs. 27G). The cytopathological examination of these samples consistently allows the identification of the melanoma cell type, confirming that the sampling for genomic testing is of a melanocytic tumor and hence guiding treatment decisions.

The principal endpoints evaluated were sufficiency of the two cytopathological aspirates obtained using the different caliber needles, quality of the sample (more or less red blood cells and acellular debris), and cytopathological assignment of melanoma cell type in the two aspirates. Descriptive analyses were performed using SPSS Statistics v22.0 (IBM, Armonk, NY, USA). The alpha error was set at 0.05.

## 3. Results

The 32 patients in this series ranged in age from 28 to 88 years (mean age 65.2 ± 13.9). Eighteen patients (56.3%) were women. The tumor was exclusively choroidal in 17 (53.1%), ciliochoroidal in 6 (18.8%), iridociliochoroidal in 7 (21.9%), and exclusively iridic in 2 (6.2%). The tumors ranged in size from 3.3 mm to 23 mm in largest basal diameter (mean LBD 13.2 ± 4.8 mm) and from 0.5 mm to 12 mm in maximal thickness (mean thickness 6.4 ± 3.3 mm). Supplemental Table S1 summarizes baseline prebiopsy characteristics of the patients and their respective tumors.

**Figure 3:**
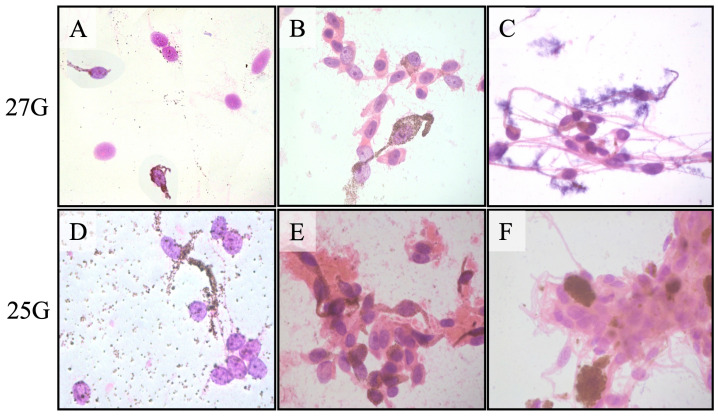
Comparison between cytology smears from fine-needle aspiration biopsy using 27-gauge and 25-gauge needles of the same tumor: (**A**) and (**D**), (**B**) and (**E**), (**C**) and (**F**). Note that the 27-gauge aspirates still yield a sufficient specimen but slight paucicellularity and with less extracellular debris.

The aspirates obtained using the different caliber needles were sufficient for cytopathological diagnosis and classification in 31 of the 32 cases (96.9%). The single case that yielded an insufficient aspirate (Patient 22) for cytopathological diagnosis using a 27-gauge needle also yielded an insufficient aspirate using the 25-gauge needle. Figure 3 shows the representative cytopathology smears using different gauge needles.

The cytopathological classification of the tumor cell type was identical in the two aspirates in all 31 cases that yielded sufficient aspirates. The obtained melanocytic tumor cells were classified as spindle melanoma cells in 14, mixed spindle and epithelioid melanoma cells in 14, epithelioid melanoma cells in 1, and nevus cells in 2. The only difference between the 25-gauge and 27-gauge aspirates was a greater amount of blood and fibrinous debris in the 25-gauge cases. Thirty-one of the 32 tumors (96.9%) also yielded sufficient aspirates for gene expression profile classification. The tumor cells were classified as Class 1 in 22 (71.0%) and Class 2 in 9 (29.0%). The three tumors classified as nevi and one tumor that yielded insufficient aspirates for cytopathological diagnosis were all classified as GEP Class 1A tumors. The tumors that yielded insufficient aspirates for cytopathological diagnosis and GEP classification were not identical: the former was a choroidal tumor 9 mm × 9 mm × 2.8 mm in size, and the latter was a ciliochoroidal tumor 16.5 mm × 15 mm × 8.2 mm in size. Table 1 depicts the final diagnosis, sufficiency of aspiration material, GEP class, and follow-up information for each patient.

The results obtained indicate that the diagnostic yield between the 25G and 27G needles was similar in most cases. However, the choice of 27G needles was driven by potential advantages in reducing complications (mostly bleeding) and improving sample quality. As predicted, the 27G needle demonstrated a lower incidence of blood contamination of the cytology specimens, which enhanced cytopathological interpretation, and we anticipate that the 27G needle improves the prognostic accuracy of the GEP as it minimizes the described issue.

## 4. Discussion

The use of FNAB has evolved to include a range of distinct approaches, each tailored to specific diagnostic and therapeutic needs. Diagnostic biopsies are critical when clinical diagnoses are uncertain, particularly in cases where malignant neoplasms are suspected [4,12]. Confirmatory biopsies, on the other hand, play a crucial role in reinforcing a diagnosis that is already highly probable, serving to reassure patients and justify complex treatments to medical peers [4]. Investigational biopsies are instrumental in assessing the efficacy of surgical techniques, tools, and diagnostic accuracy, as well as in evaluating the safety and prognostic potential of various treatment approaches [4]. Lastly, prognostic biopsies have become a cornerstone in determining the future course of a disease, especially for melanocytic uveal tumors, where they assist in classifying tumors for prognostic purposes using validated laboratory methods [1,3,13,14]. It is well stablished that uveal melanomas GEP Class II have a greater probability of spreading clinical metastasis in the affected patient compared to the GEP Class I [14].

Each biopsy type, with its unique purpose and methodology, significantly contributes to the nuanced and precise management of intraocular tumors, underscoring the importance of tailored approaches in medical diagnostics and treatment planning [1,4,12].

Our results demonstrated that both 25G and 27G FNAB needles yield sufficient material for cytopathological and GEP classifications of uveal melanocytic tumors. To the best of our knowledge, this is the largest study, albeit retrospective, to analyze the results from specimens acquired using two different needle gauges with a standardized FNAB technique for uveal melanocytic tumors.

Klofas LK et al. (2021) [6] has also reported an equivalent success rate (75%, 6 out of 8 patients) for in vivo samples between 25G and 27G needles while consistently using a transscleral technique. Their much smaller cohort was also supported by a retrospective review of 100 cases using series of in vitro, ex vivo, and in vivo studies to assess biopsy yield. Similarly, our results corroborate their findings showing the same sufficiency material rate between 25G and 27G lumens in over 95% of the cases comparing the same patients and using the same technique by the same surgeon. Our group has shown a slightly higher cellular yield rate for the 25G needle (99.4%, 158/159) [3], and Klofas et al. showed a perfect success using the 27G needle (100%, 65/65) [6] while obtaining sufficient material for cytopathological and gene profile classification. The cohort reported by Klofas et al. included a mix of different techniques and 8 enucleated eyes with large tumors. Our study shares real-life, clinical cases of patients that retained their eyes. Of note, an insufficient material result can also be informative, as more cohesive tumors are associated with more differentiation and perhaps a higher likelihood of being benign [5]. Such an understanding drove us to investigate the biopsy yield of 25G and 27G needles in consecutive patients, using different biopsy routes and including clinically diagnosed uveal melanomas as well as indeterminate small lesions. These measures likely minimized multiple biases and made this small cohort representative of real-world clinical practice.

It is relevant to highlight that a smaller gauge potentially minimizes the risk of retinal traction and detachment, addressing key concerns associated with FNAB. These benefits, coupled with the comparable diagnostic sufficiency of 25G needles, motivated the transition to 27G needles in our institutional practice.

This study has several limitations. First, its retrospective design limits control over confounding variables. Second, while this is one of the largest comparative studies of 25G vs. 27G FNAB needles in uveal melanoma, the sample size remains small for detecting rare biopsy failures. Third, the study design involved performing GEP analysis exclusively with the 27G needles since our group has shown that GEP testing requires a substantially small sample compared to cytology [3], and the authors believed the aspirated yield would be better assessed directly under the microscope [3,9,10]. Finally, all procedures were performed by an experienced ocular oncologist in a controlled setting, which may limit generalizability among less experienced surgeons.

## 5. Conclusions

This study demonstrates that FNAB using 27G needles provides a diagnostic yield comparable to 25G needles while offering potential advantages in reducing blood contamination and procedural complications. The transition to 27G was guided by its ability to improve sample quality, minimize vitreous hemorrhage, and reduce retinal traction without compromising cytopathological sufficiency. Given our findings, 27G FNAB needles may be considered a sustainable alternative to 25G needles for biopsies of melanocytic uveal tumors. Future studies with larger sample sizes and long-term follow-up will be able to validate these findings and refine biopsy protocols in ocular oncology.

## Figures and Tables

**Table 1:** Conclusive diagnosis, gene expression profile, and subsequent follow-up data for 32 patients using diagnostic fine-needle aspiration biopsy.

Patient	Adequacy of the Aspirate	Melanoma Cell Type	Final Dx	Initial Management	GEP Class	Metastasis	OS (Months)	Life Status
1	Yes	Mixed	UM	Enucleation	1A	No	23	Living
2	Yes	Spindle	UM	I-125	2	No	14	Living
3	Yes	Epithelioid	UM	Enucleation	2	Yes	93	Living
4	Yes	Mixed	UM	I-125	2	Yes	39	Deceased
5	Yes	Mixed	UM	I-125	2	No	11	Living
6	Yes	Mixed	UM	Enucleation	1A	No	50	Deceased
7	Yes	Spindle	UM	I-125	1A	No	14	Living
8	Yes	Spindle	UM	I-125	1A	No	23	Living
9	Yes	Spindle	UM	I-125	1A	No	23	Living
10	Yes	Mixed	UM	I-125	2	Yes	3	Deceased
11	Yes	Mixed	UM	I-125	Failed	No	23	Living
12	Yes	Spindle	UM	I-125	1A	No	94	Living
13	Yes	Spindle	UM	Resection	1A	No	26	Living
14	Yes	Mixed	UM	Enucleation	1A	No	27	Living
15	Yes	Mixed	UM	Plaque	2	Yes	52	Deceased
16	Yes	Spindle	UM	Plaque	1A	No	31	Living
17	Yes	Spindle	UM	Plaque	2	No	62	Living
18	Yes	Mixed	UM	Plaque	1A	No	26	Living
19	Yes	Spindle	UM	Plaque	1A	Yes	52	Deceased
20	Yes	Mixed	UM	Plaque	1B	No	51	Living
21	Yes	Mixed	UM	Enucleation	2	No	12	Living
22	No *	QNS	UN	Observation	1A	No	2	Living
23	Yes	Spindle	UM	Plaque	1B	Yes	86	Deceased
24	Yes	Spindle	UM	Enucleation	1A	No	96	Living
25	Yes	Spindle	UM	Plaque	1A	No	96	Living
26	Yes	Spindle	UM	Plaque	1A	No	9	Living
27	Yes	Mixed	UM	Plaque	1A	Yes	6	Deceased
28	Yes	Mixed	UM	Plaque	1A	No	52	Living
29	Yes	Nevus	UN	Observation	1A	No	0	Living
30	Yes	Nevus	UN	Observation	1A	No	64	Living
31	Yes	Mixed	UM	Plaque	1A	No	70	Living
32	Yes	Spindle	UM	Plaque	1A	No	15	Deceased

QNS: quantity not sufficient; * specimen inadequate when obtained by 25G and 27G needles; Melanoma Cell Type: cytology result; Final Dx: final diagnosis based on biopsy findings; UM: uveal melanoma; UN: uveal nevus; management: initial treatment based on biopsy result; Plaque: I-125 brachytherapy; GEP: Gene Expression Profile Classification; OS: overall survival.

## Data Availability

All data generated and analyzed during this study are included in this article. Further inquiries can be directed to the corresponding author.

## Supplementary Materials

The following supporting information can be downloaded at: https://www.mdpi.com/article/10.3390/jcm14113650/s1; Table S1: Baseline prebiopsy characteristics of the 32 patients and their respective tumors.
