# Supplementary material for: Comparison of Cytopathology Yield of Fine-Needle Aspiration Biopsy Using 25G Versus 27G Needles for Melanocytic Uveal Tumors
Source: J Clin Med. 2025 May 23;14(11):3650. doi: 10.3390/jcm14113650 (PMC12155546; doi:10.3390/jcm14113650)
Supplement: Supplementary file 1 [file jcm-14-03650-s001.zip › jcm-3330857-supplementary.pdf]

Supplemental Table S1. Baseline prebiopsy characteristics of the 32 patients their respective tumors.

Patient	Age	Sex	BVCA (Snellen)	Ciliary Body Involvement	Tumor Size (mm)			Location: Distance from (mm)		Clinical Dx	FNAB route
					Largest Basal Diameter	Smallest Basal Diameter	Maximal Thickness	Optic Disc	Macula		
1	57	M	HM	N	15.0	14.5	12.0	0.0	0.0	UM	TV
2	83	F	20/50	Y	18.0	17.0	11.0	10.5	13.5	UM	TS
3	58	F	20/50	Y	18.5	17.5	11.5	8.5	9.5	UM	TS
4	68	F	20/40	Y	14.0	13.0	9.4	17.0	14.0	UM	TS
5	68	F	20/60	Y	19.5	19.0	11.0	6.0	9.5	UM	TS
6	78	F	NLP	Y	16.0	9.0	3.5	23.0	23.0	UM	TS
7	61	M	20/20	N	12.5	12.0	6.1	7.5	3.5	UM	TV
8	84	F	20/40	N	6.0	5.3	4.5	0.2	4.5	UM	TV
9	54	F	20/20	N	11.0	10.0	5.3	7.0	3.5	UM	TV
10	75	M	20/25	Y	14.0	12.0	7.1	16.5	15.0	UM	TS
11	64	F	20/30	Y	16.5	15.0	8.2	7.5	4.0	UM	TS
12	73	M	20/40	N	11.0	10.0	7.2	10.0	8.0	UM	TV
13	53	F	20/20	N	3.3	3.3	1.2	23.0	23.0	UN vs. UM	TA
14	62	M	20/80	Y	23.0	18.0	5.8	5.0	6.0	UM	TV
15	76	M	20/200	N	17.0	15.0	7.0	0.4	0.0	UM	TV
16	28	F	20/80	Y	14.0	13.0	5.7	8.0	12.5	UM	TS
17	74	F	20/40	N	14.0	9.5	3.6	4.5	9.0	UM	TV
18	79	M	20/50	Y	17.5	17.0	11.0	7.5	6.5	UM	TS
19	85	M	20/100	Y	12.0	9.5	7.6	13.0	8.5	UM	TS
20	58	F	CF	N	13.5	10.5	5.5	0.0	0.0	UM	TV
21	76	F	20/40	Y	15.0	13.0	8.6	12.5	11.0	UM	TS

22	62	F	20/30	N	9.0	9.0	2.8	8.5	10.5	UN vs. UM	TV
23	57	M	CF	N	12.0	13.0	10.8	5.0	5.0	UM	TV
24	57	M	20/100	Y	18.5	10.0	8.0	9.0	10.5	UM	TS
25	58	M	20/25	N	10.0	8.5	3.2	6.5	4.0	UM	TV
26	63	M	20/50	N	6.0	5.3	1.8	0.0	0.0	UM	TV
27	88	M	20/300	N	20.0	17.5	9.0	0.5	0.2	UM	TS
28	57	F	20/20	N	8.5	8.5	4.0	1.0	5.0	UN vs. UM	TV
29	63	F	20/20	N	9.0	8.0	2.2	8.5	7.0	UN vs. UM	TV
30	29	F	20/15	N	3.5	2.0	0.5			UN vs. UM	TA
31	60	M	20/25	N	10.0	9.0	7.5	4.5	7.0	UM	TV
32	80	F	20/40	N	13.0	12.0	2.5	2.5	0.0	UM	TV

M: Male; F: Female; BVCA: Best Visual Corrected Acuity of the Affected Eye (Snellen at 20 feet); CF: Count Fingers; N: No; Y: Yes; Clinical Dx: clinical diagnosis; UM: uveal melanoma; UN: uveal nevus; FNAB route: needle route used for biopsy; TV: transvitreal, TS: transscleral; TA: transaqueous.
